# *Salvia miltiorrhiza* Alleviates Memory Deficit Induced by Ischemic Brain Injury in a Transient MCAO Mouse Model by Inhibiting Ferroptosis

**DOI:** 10.3390/antiox12040785

**Published:** 2023-03-23

**Authors:** Geon Ko, Jinho Kim, Yeong-Jae Jeon, Donghun Lee, Hyeon-Man Baek, Keun-A Chang

**Affiliations:** 1 Department of Health Sciences and Technology, GAIHST, Gachon University, Incheon 21999, Republic of Korea; 2 Department of Herbal Pharmacology, College of Korean Medicine, Gachon University, Seongnam-si 13120, Republic of Korea; 3 Department of Molecular Medicine, College of Medicine, Gachon University, Incheon 21999, Republic of Korea; 4 Department of Pharmacology, College of Medicine, Gachon University, Incheon 21999, Republic of Korea; 5 Department of Basic Neuroscience, Neuroscience Research Institute, Gachon University, Incheon 21999, Republic of Korea

**Keywords:** stroke, transient middle cerebral artery occlusion, brain injury, cognitive impairment, *Salvia miltiorrhiza*

## Abstract

*Salvia miltiorrhiza* (SM) has been used in oriental medicine for its neuroprotective effects against cardiovascular diseases and ischemic stroke. In this study, we investigated the therapeutic mechanism underlying the effects of SM on stroke using a transient middle cerebral artery occlusion (tMCAO) mouse model. Our results showed that SM administration significantly attenuated acute brain injury, including brain infarction and neurological deficits, 3 days after tMCAO. This was confirmed by our magnetic resonance imaging (MRI) study, which revealed a reduction in brain infarction with SM administration, as well as our magnetic resonance spectroscopy (MRS) study, which demonstrated the restoration of brain metabolites, including taurine, total creatine, and glutamate. The neuroprotective effects of SM were associated with the reduction in gliosis and upregulation of inflammatory cytokines, such as interleukin-6 (IL-6) and Tumor necrosis factor-α (TNF-α), along with the upregulation of phosphorylated STAT3 in post-ischemic brains. SM also reduced the levels of 4-Hydroxynonenal (4-HNE) and malondialdehyde (MDA), which are markers of lipid peroxidation, induced by oxidative stress upregulation in the penumbra of the tMCAO mouse brain. SM administration attenuated ischemic neuronal injury by inhibiting ferroptosis. Additionally, post-ischemic brain synaptic loss and neuronal loss were alleviated by SM administration, as demonstrated by Western blot and Nissl staining. Moreover, daily administration of SM for 28 days after tMCAO significantly reduced neurological deficits and improved survival rates in tMCAO mice. SM administration also resulted in improvement in post-stroke cognitive impairment, as measured by the novel object recognition and passive avoidance tests in tMCAO mice. Our findings suggest that SM provides neuroprotection against ischemic stroke and has potential as a therapeutic agent.

## 1. Introduction

Stroke is a life-threatening disease characterized by the rapid development of clinical signs of focal or global impairment of cerebral function, with symptoms persisting for 24 h or up to weeks, or leading to death with no apparent cause other than a vascular origin [1]. There are over 12.2 million new strokes each year [2]. Over 16% of all strokes occur in people 15–49 years of age and over 62% of all strokes occur in people under 70 years of age [3]. Depending on where the stroke occurs in the brain, it leads to various symptoms, such as hemiparesis, speech disorder, movement disorder, and altered consciousness, and can cause serious sequelae or can be life-threatening [4]. After stroke onset, 44% of patients suffer from memory-related deficits, such as global cognitive impairment, decreased attention and processing speed, and frontal executive function deficits [5]. Moreover, 88% of stroke patients have hemiparesis-like muscle weakness or one-sided partial paralysis in the lower and upper limbs [6]. Although tissue plasminogen activator and mechanothrombectomy is used to treat ischemic stroke, it must be used within 4.5 h of stroke onset because of the side effects, such as hemorrhages. Neurological dysfunction occurs in stroke because of a sudden interruption of blood flow to the brain, resulting in sustained brain damage through diverse pathogenic mechanisms, including excitotoxicity, oxidative stress, and programmed cell death [7].

Ferroptosis is a form of regulated cell death that is characterized by the accumulation of lipid peroxidation and iron-dependent reactive oxygen species (ROS) in cells [8]. Recent studies have suggested that ferroptosis may play a role in the pathophysiology of a stroke, a condition that occurs when blood flow to the brain is disrupted, leading to brain cell death. Studies have shown that ferroptosis contributes to the secondary injury cascade following stroke [9]. During a stroke, the disruption of blood flow leads to a decrease in oxygen and glucose supply to the brain, which in turn leads to a buildup of ROS and lipid peroxides in the affected brain tissue [10,11]. This accumulation of ROS and lipid peroxides triggers ferroptosis, which leads to further cell death and exacerbates the damage caused by the stroke [12]. Additionally, studies have shown that targeting ferroptosis may be a potential therapeutic approach for stroke [13]. Drugs that inhibit lipid peroxdiation or ROS production have been shown to have neuroprotective effects in animal models of stroke [14,15]. For example, the drug Ferrostatin-1, which inhibits lipid peroxidation, has been shown to reduce brain damage and improve neurological function in animal models of stroke [16]. Therefore, ferroptosis appears to play a significant role in the pathophysiology of stroke and may be a promising target for the development of new therapeutic interventions.

Recently, there has been intense interest in the antioxidant properties of natural products owing to their neuroprotective effects in stroke patients [17,18]. Among these natural products, *Salvia miltiorrhiza* (SM) is widely used to treat vascular diseases, especially ischemic cardiovascular diseases [19,20,21]. Experimental studies have shown that SM dilates the coronary arteries, increases coronary blood flow, and scavenges free radicals in ischemic diseases, thereby reducing cellular damage from ischemia and improving cardiac functions [22,23]. In various diseases, such as myocardial ischemia, atherosclerosis, hyperlipidemia, and Alzheimer’s disease, SM suppresses oxidative stress and inflammation and shows neuroprotective effects. Although the mechanisms of action of SM may be related to its anticoagulant, antioxidant, anti-inflammatory, and apoptosis-inhibitory effects [24], the therapeutic mechanism of SM on ischemic stroke remains to be elucidated. Therefore, the purpose of this study was to investigate the therapeutic mechanism of SM for ischemia/reperfusion (I/R) injury of cerebral ischemia in transient middle cerebral artery occlusion (tMCAO) mice. We first investigated whether SM could exert neuroprotective effects against acute brain injury following tMCAO by assessing its effects on brain infarction and neurological deficits 3 days after tMCAO. The infarct volume was assessed using magnetic resonance imaging (MRI) and 2,3,5-Triphenyltetrazolium chloride (TTC) staining. After administering SM repeatedly for 28 days following tMCAO, we assessed its neuroprotective effects against brain injury by evaluating its impact on neurological deficits, survival rates, and cognitive impairment. We further investigated the therapeutic mechanisms underlying the long-term neuroprotective effects of SM in a tMCAO stroke model.

## 2. Materials and Methods

### 2.1. Animal

Healthy male ICR mice (33 ± 3 g) (DBL Co, Ltd., Eumseong-gun, Chungbuk, Republic of Korea) were kept in a facility system under controlled temperature (22 ± 2 °C), humidity (50 ± 10%), and automatic day–night rhythm (12 h-cycle). All mice were provided with water and food ad libitum. Prior to the experiment, mice were allowed a 1-week adaptation. All animal experiments were approved by the Institutional Animal Care and Use Committee of Lee Gil Ya Cancer and Diabetes Institute, Gachon University (LCDI-2021-0078).

### 2.2. Induction of tMCAO in Mice

Focal ischemic stroke was induced in 10-week-old male ICR mice using the MCAO method, as described previously [25]. Animals were anesthetized with isoflurane (3% induction; 1.5% maintenance with medical O_2_). Throughout the surgical period, the body temperature of the animals was maintained within the normal range using a heating pad. The laser Doppler flowmeter probe (PeriFlux 6000 system, PERIMED, Järfälla, Sweden) was attached to the skull (2 mm posterior to the bregma and 5–6 mm laterally) and blood flow obstruction was monitored during tMCAO induction (Figure S1). Next, the right carotid region was exposed, and the external and common carotid arteries were ligated with a 6-0 monofilament (Doccol Corp., Sharon, MA, USA). A 20-mm-length 6-0 silicon-coated monofilament was introduced through the external carotid artery, occluding the MCA origin. After 90 min of occlusion, reperfusion was permitted by the gentle withdrawal of the monofilament (under the same anesthetic conditions during surgery). The control animals underwent sham surgery without suture insertion. The incision was sutured, and the mice were placed in a warm recovery cage. The animals were returned to their cages and provided free access to food and water.

### 2.3. Experimental Groups and Drug Treatment

All mice were randomized into three different treatment groups: Group 1, sham-operated animals without MCAO and treated with the control vehicle only (0.9% normal saline) per oral (sham; *n* = 9); Group 2, animals with tMCAO and treated with the control vehicle p.o. (tMCAO-V; *n* = 8); and Group 3, animals with tMCAO and 200 mg/kg SM treatment p.o. (tMCAO-SM; *n* = 13). The administration volume was 0.05 mL/10 g for body weight. The first administration was performed on the same day of injury within 1 h after reperfusion. SM was orally administrated once daily for 3 or 28 days, including the first administration within 1 h after reperfusion.

The roots of SM were purchased from Yaksudang Pharmaceutical Co. Ltd. (Seoul, Republic of Korea). A voucher specimen (2009150001) was deposited by Professor Donghun Lee of Herbal Pharmacology, College of Korean Medicine, Gachon University. The SM was extracted from 30% ethanol for 3 h at 85 °C in a reflux apparatus. The extract was filtered and concentrated under reduced pressure and freeze-dried at −80 °C. The yield of the extract was 37.4%. Chromatographic analysis of SM was performed by HPLC linked using an 1100 series HPLC system (Agilent, Santa Clara, CA, USA) (Figure S2). The SM extract was dispersed in 0.9% normal saline (vehicle) at a concentration of 40 mg/ ml immediately before use. This mixture was orally administered to mice at a concentration of 200 mg/kg, which was selected based on previous results [26].

### 2.4. Neurological Deficit Scoring (mNSS) and Survival Rate

Neurological deficits were assessed daily at 24 h after tMCAO according to a Modified Neurological Severity Score (mNSS), in which motor, sensory, balance, and reflex tests were assessed using an 18-point scale (normal = 0; maximal deficit score = 18) [27]. In the severity scores of injuries, a score of 1 was awarded for the inability to perform the test or the lack of a tested reflex; thus, the higher the score, the more severe the injury [27]. All neurological scoring tests were performed using the blind method, with the observers blinded to the treatments administered to the animals. In addition to neurological assessment, the survival rate of the mice was determined by calculating the percentage of dead mice during the experimental time course.

### 2.5. Quantification of Infarction Size

In ischemic stroke, neuronal cells that receive insufficient blood from blocked vessels are damaged. To quantify injury, brain tissue was stained with TTC dissolved in 0.9% normal saline. A regular healthy hemisphere (contralateral hemisphere) was used to set the standard volume of the brain. The opposite hemisphere (ipsilateral hemisphere), except for the damaged area, was measured, and the degree of damage was calculated as follows: (contralateral hemispheric healthy area in the ipsilateral hemisphere)/(contralateral hemisphere) %).

### 2.6. Neuroimaging

All mice were examined using a BioSpec 9.4T MRI system (Bruker BioSpin Corporation, Billerica, MA, USA) at the Core-Facility for Cell to In Vivo Imaging. MRI was performed at 3 and 10 d after surgery. Each mouse was deeply anesthetized and placed in the supine position, with its head in the middle of the coil. Throughout the entire MR study, the animals were maintained in an anesthetized state with 2.0–2.5% isoflurane and a 1:2 mixture of O_2_ and NO_2_ (250 mL/min) according to respiratory rate; respiration was monitored; and the temperature was maintained by circulating warm water during scans.

Multislice T2-weighted images were acquired using the tube rapid acquisition with relaxation enhancement (RARE) technique [28], echo time (TE) = 17.54 ms and repetition time (TR) = 5000 ms, effective TE = 33 ms, RARE factor = 8, average = 4, field of view (15×15 mm^2^, matrix size = 150 × 150, and slice thickness = 0.50 mm to locate the volume of interest (VOI) in the left and right striatum (8 μL). Thereafter, localized shimming was performed to improve the field homogeneity in the VOI. After shimming, localized ^1^H MR spectrum was obtained using a point-resolved spectroscopy [29] technique with TE = 17.54 ms, TR= 5000 ms, 320 averages, data points = 2048, spectral width = 4401.41 Hz, and VOI size = 2 × 2 × 2 mm^3^ in combination with outer volume suppression and VAPOR water suppression [30]. In vivo ^1^H-MR spectra were analyzed using a linear combination analysis method, LCModel [31], and absolute quantification was performed. Lesion volumes were also evaluated from T2-weighted images as the ratio of the ischemic region to the corresponding region in the contralateral hemisphere (mean ± standard deviation). A *p*-value of < 0.05 was considered statistically significant (* *p* < 0.05; ** *p* < 0.01; *** *p* < 0.001; and **** *p* < 0.0001) by Tukey’s multiple comparison tests.

### 2.7. Behavior Test

#### 2.7.1. Novel Object Recognition (NOR) Test

Three weeks after tMCAO, the NOR test was performed on 3 consecutive days. On the first day, a spatial adaptation experiment was conducted in which the tMCAO mice freely explored a square space (40 cm × 40 cm × 40 cm) with four black sides for 10 min. The following day, two identical objects were placed diagonally opposite to each other in the box, and the mouse was free to explore the objects in the box for 10 min. On day 3, one of the same objects was replaced with a new one, and while the mouse freely explored the objects in the box for 10 min, it was recorded with a charge-coupled device camera (SCB-3000, hanhwa aerospace, Changwon, Republic of Korea) camera and analyzed using a motion analysis program (Ethovision XT 9.0 system, Noldus, Wageningen, The Netherlands).

#### 2.7.2. Passive Avoidance Test (PAT)

A PAT was performed for 3 continuous days using a passive avoidance apparatus (42.5 cm wide and 35.5 cm long, Gemini Passive Avoidance System; San Diego Instruments, San Diego, CA, USA), composed of two adjacent bright and dark chambers connected by a remote operational gate. On the first day, tMCAO mice were allowed to freely explore the chambers for 5 min. The following day, when the tMCAO mouse entered the dark chamber by crossing the sliding door, it was exposed to an electric shock to the feet at 0.3 mA for 5 s with the gate closed. After 24 h, the latency time for the mice to enter the dark chamber from the bright chamber was measured.

### 2.8. Tissue Processing and Molecular Works

#### 2.8.1. Tissue Preparation

The mice were anesthetized with a mixture of Zoletil (8.3 mg/kg) and Rompun (15 mg/kg), and their brains were extracted. For Western blot (WB) analysis, the penumbra region (including cortex and striatum) which is a boundary between healthy and core region was dissected from the ipsilateral hemisphere by TTC staining and immediately frozen in liquid nitrogen. The contralateral hemisphere of the mouse’s brain was also immediately frozen in liquid nitrogen. For immunohistochemistry, the brain tissue was fixed in 4% paraformaldehyde at 4 °C for 24 h and osmoprotected with 30% sucrose solution for 3 days. Brains were frozen in molds filled with optimal cutting temperature compounds (Sakura, Osaka, Japan). Frozen tissues were cut into 30 μm thickness using a cryomicrotome (Cryotome, Thermo Electron Corporation, Waltham, MA, USA) and stored in a cryoprotectant solution (ethylene 30% and glycerol 30% in Phosphate buffer saline (PBS)) at 4 °C.

#### 2.8.2. Western Blot (WB)

The penumbra area identified by TTC staining (including cortex and striatum) was lysed with Radioimmunoprecipitation assay buffer (RIPA) buffer (150 mM NaCl, 1% NP-40, 0.5% sodium deoxycholate, 0.1% sodium monododecyl sulfate (SDS, 50 mM Tris, pH 8.0) containing protease inhibitors (Roche Applied Science, Mannheim, Germany) and a cocktail of phosphatase inhibitors (Sigma-Aldrich, St. Louis, MO, USA) on ice for 30 min. After centrifugation at 13,000 revolutions per minute (RPM) for 20 min at 4 °C, the lysates were quantified using the Bradford assay solution (Bio-Rad Laboratories, Inc., Hercules, CA, USA). Appropriate amounts of protein were loaded onto 8% or 15% sodium dodecyl sulfate-polyacrylamide gel electrophoresis gels. After being transferred onto a polyvinylidene difluoride membrane (Merck, Kenilworth, NJ, USA), the transferred membrane was incubated with a blocking buffer (5% skim milk or 3% Bovine serum albumin (BSA) in TBS-T at room temperature for 1 h and then incubated with the appropriate primary antibody diluted in the TBS-T solution containing 3% bovine serum albumin overnight at 4 °C. After washing three times with TBS-T, the membrane was incubated with the appropriate secondary antibody for 1 h at room temperature. Protein bands were detected using Enhanced Peroxidase Detection enhanced chemiluminescence (ELPISBIO, Daejeon, Republic of Korea) or Immobilon Western Chemiluminescent HRP Substrate (Millipore, Burlington, MA, USA). Quantification of the bands was performed using ImageJ software v1.4.3.67 (*n* = 4–6 per group).

The primary antibodies used in this experiment were the following: GFAP (1:5000, DAKO, Fort Collins, Centennial, CO, USA), Iba-1 (1:2000, Novus, Centennial, CO, USA), pSTAT3 (1:2000, Santacruz, Dallas, TX, USA), STAT3 (1:2000, Santacruz, Dallas, TX, USA), iNOS (1:4000, BD Biosciences, San Jose, CA, USA), 4-HNE (1:2000, R&D Systems, Minneapolis, MN, USA), β-actin (1:3000, Santacruz, Dallas, TX, USA), FPN1 (1:3000, Alpha Diagnostic, San antonio, TX, USA), glutathione peroxidase 4 (GPX4) (1:3000, Abcam, Waltham, MA, USA; Boston, MA, USA), acyl-CoA synthetase long-chain family member 4 (ACSL4) (1:2000, Abcam, Waltham, MA, USA; Boston, MA, USA), PSD-95 (1:3000, Thermo Fisher, Waltham, MA, USA), and synaptophysin (1:5000, Abcam, Waltham, MA, USA; Boston, MA, USA).

#### 2.8.3. Immunohistochemistry (IHC)

To confirm changes in gliosis, immunofluorescence analysis was performed as previously described [32]. After washing in PBS-T (0.4% Triton X-100 in PBS), brain sections were blocked in blocking solution (1% BSA and 3% normal goat serum in 0.4% PBS-T) at room temperature for 1 h. Then, slices were incubated overnight at 4 °C in PBS-T solution with the following primary antibodies: mouse anti-GFAP (1:500, DAKO, Centennial, CO, USA) and rabbit anti-Iba1 (1:500, Sigma, St. Louis, MO, USA). After washing in PBS-T, the tissues were incubated with Alexa Fluor 594-conjugated goat anti-rabbit (1:500, Invitrogen, Carlsbad, CA, USA) or Alexa Fluor 488-conjugated goat anti-mouse (1:500, Invitrogen) antibodies for 1 h at room temperature, with DAPI as a counterstain.

All images were captured using a Nikon TS2-S-SM microscope (Nikon Microscopy, Tokyo, Japan) equipped with a Nikon DS-Qi2 camera and further analyzed using ImageJ 1.50 software (National Institutes of Health, Bethesda, MD, USA). The penumbra area (including striatum and cortex) was selected as a region of interest for IHC, the intensity of the fluorescence signal was measured and converted into a percentage (*n* = 3–4 mice per group).

#### 2.8.4. Enzyme-Linked Immunosorbent Assay (ELISA)

The levels of IL-6, TNF-α, MDA, and GSH were analyzed using commercial ELISA kits according to the manufacturer’s instructions (IL-6: R&D Systems, Minneapolis, MN, USA, DY406-05; GSH: Cayman, Ann Arbor, MI, USA, 703002; TNF-α: R&D Systems, Minneapolis, MN, USA, DY410-05; MDA: BioVision, Waltham, MA, USA, k739-100). The protein levels were confirmed in duplicate and measured using a VICTOR X4 Multimode Plate Reader (PerkinElmer, Waltham, MA, USA).

#### 2.8.5. Nissl Staining

Nissl staining was performed using a Cresyl violet acetate solution. The brain sections were washed with PBS to remove the anti-freezing solution and dried for an hour. Brain sections were immersed in 0.5% Cresyl violet stain solution for 10 min. The immersed brain sections were washed with H_2_O for 10 s and with 70%, 95%, and 100% ethanol continuously for 1 min. Finally, brain sections were immersed in xylene for 5 min. Brain sections were placed on a glass slide. A cover glass was mounted on the sections.

### 2.9. Statistical Analysis

All statistical analyses and outlier removal (significance level: alpha = 0.05) were performed using GraphPad Prism version 8.2.1 software (GraphPad Software Inc., San Diego, CA, USA). All graph data were marked as mean ± standard error (SEM). The data collected from the NOR memory index between the groups were analyzed using a two-way analysis of variance (ANOVA), followed by Bonferroni’s multiple comparison test. Data collected from PAT, IHC, WB, and ELISA were analyzed by one-way ANOVA followed by Tukey’s multiple comparison test. The mNSS scoring, TTC staining, and MRI/magnetic resonance spectrometry (MRS) data of the vehicle- and SM-treated groups were analyzed using an unpaired *t*-test. Differences in survival rates between the groups were analyzed using the log-rank (Mantel–Cox) test. Statistical significance was set at a *p*-value of <0.05 (* *p* < 0.05; ** *p*< 0.01; *** *p* < 0.001; and **** *p* < 0.0001).

## 3. Results

### 3.1. SM Administration Reduced Cerebral I/R Injury and Recovered Metabolites in tMCAO Mouse Brain

**Figure 1:**
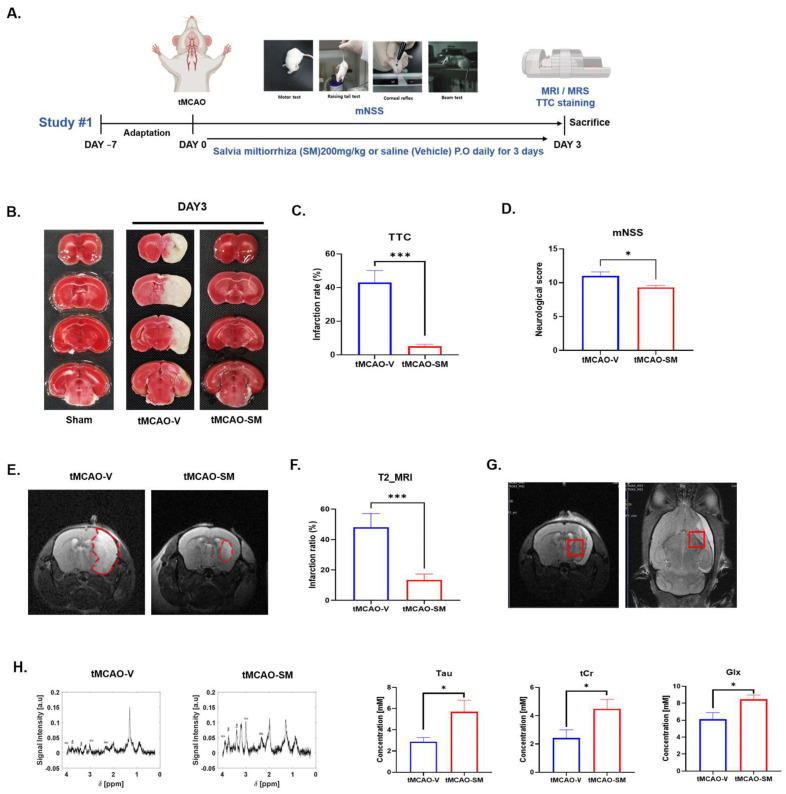
SM administration reduced cerebral I/R injury and recovered metabolites in tMCAO mouse brain. (**A**) Experimental scheme. (**B**) TTC staining and (**C**) quantification of infarction ratio between tMCAO-V and tMCAO-SM group. (**D**) mNSS score. (tMCAO-V, *n* = 7; tMCAO-SM, *n* = 7) (**E**) Representative image of penumbra area (Red dotted) in tMCAO-V and tMCAO-SM groups and (**F**) quantification of penumbra area in MRI T2 imaging. (tMCAO-V, *n* = 8; tMCAO-SM, *n* = 12) (**G**) Signal intensity of magnetic resonance spectroscopy between tMCAO-V and tMCAO-SM. (**H**) Metabolites analysis between tMCAO-V and tMCAO-SM group. Values are expressed as the mean ± SEM. (Tau, tMCAO-V, *n* = 7; tMCAO-SM, *n* = 7; Cr, tMCAO-V, *n* = 8; tMCAO-SM, *n* = 7; Glx, tMCAO-V, *n* = 8; tMCAO-SM, *n* = 6) Statistical analysis between the two groups was performed using unpaired *t* test. * *p* < 0.05 and *** *p* < 0.001.

To determine the protective effects of SM against acute brain injury in mice after MCAO and reperfusion, brain infarction was quantified 3 days after tMCAO (Figure 1A). tMCAO mice administered 200 mg/kg SM daily (tMCAO-SM) showed a reduced cerebral infarction volume compared to tMCAO-V mice (Figure 1B). Compared to tMCAO-V mice (43.09 ± 6.99), the reduction in infarction volume in tMCAO-SM mice (5.14 ± 1.15, *** *p* < 0.001) over 3 days was reduced to about 43% (Figure 1B). Additionally, tMCAO-SM mice (4.27 ± 1.81, * *p* < 0.05) exhibited a significant reduction in brain edema compared to tMCAO-V mice (17.52 ± 4.43) (Figure S3). In the MRI on day 3 of the stroke onset, the infarction ratio (red dotted, penumbra area) in the tMCAO-SM group (13.37 ± 3.97, *** *p* < 0.001) was significantly reduced compared to the tMCAO-V group (48.03 ± 9.00) (Figure 1C). Additionally, MRS shows significantly increased taurine (Tau), total creatine (tCr), and glutamine/glutamate (Glx) concentration among several brain metabolites in the tMCAO-SM group (Tau, 5.69 ± 1.08; tCr, 4.47 ± 0.67; Glx, 8.46 ± 0.51; * *p* < 0.05) than those in the tMCAO-V group (Tau, 2.86 ± 0.40; tCr, 2.41 ± 0.58; Glx, 6.11 ± 0.78) (Figure 1D,E).

After SM was administrated for 10 days, I/R injury and changes in brain metabolites were investigated through MRI/MRS and TTC analyses (Figure S4A). On MRI T2 imaging, I/R injury was reduced by SM administration for 10 days, but not significantly (Figure S4B). In the MRS results, changes in brain metabolites were not different in the two tMCAO groups (Figure S4C). However, the infarct rate in the tMCAO-SM group was significantly decreased compared to in the tMCAO-V group (Figure S4D).

### 3.2. SM Administration Reduced Gliosis in tMCAO Mouse Brain

**Figure 2:**
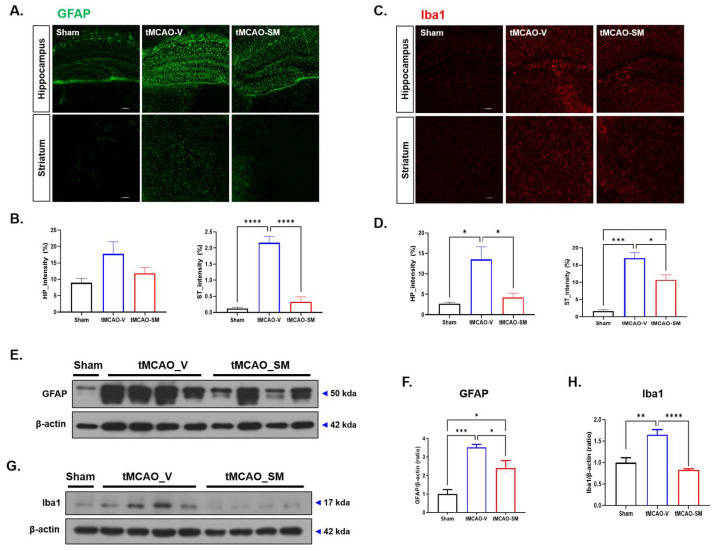
SM alleviates gliosis in tMCAO mouse brain. (**A**) Representative image of GFAP staining in hippocampus and striatum. (**B**) Quantification of interest of GFAP immunofluorescence in hippocampus and striatum. Scale bars, 100 μm. (Sham, *n* = 3; tMCAO-V, *n* = 6; tMCAO-SM, *n* = 7) (**C**) Representative image of Iba1 staining in hippocampus and striatum. (**D**) Quantification of interest of Iba-1 immunofluorescence in hippocampus and striatum. (Sham, *n* = 3; tMCAO-V, *n* = 4; tMCAO-SM, *n* = 4). (**E**) Western blot to confirm GFAP expression level in penumbra area including cortex and striatum. (**F**) Quantification of GFAP level in Western blot. (**G**) Western blot to confirm Iba1 expression level in penumbra area including cortex and striatum. (**H**) Quantification of Iba1 level in Western blot. β-actin is a loading control. Values are expressed as mean ± SEM (Sham, *n* = 4; tMCAO-V, *n* = 6; tMCAO-SM, *n* = 6). Statistical analysis between the three groups was performed using the one-way ANOVA followed by Tukey’s multiple comparisons test. * *p* < 0.05, ** p < 0.01, *** p < 0.001, and **** p < 0.0001.

Inflammation is a diverse pathogenic mechanism underlying neural cell death during the acute and chronic phases of cerebral ischemia [33]. Therefore, we determined whether SM administration could attenuate gliosis in the penumbra area of tMCAO mouse brains, as evidenced by the decreased numbers of GFAP-positive astrocytes and Iba1-positive microglia at 3 days after tMCAO (Figure 2A–D). In the penumbra area of the hippocampus, GFAP-positive cells were reduced more in the tMCAO-SM group (8.73 ± 1.74) than those in the tMCAO-V group (15.16 ± 2.86). Interestingly, in the penumbra area of the striatum, the number of GFAP-positive cells was significantly reduced in tMCAO-SM (0.35 ± 0.09, **** *p < 0.0001)* when compared to tMCAO-V (1.54 ± 0.41) (Figure 2A,B). In addition, in the penumbra area of the hippocampus, the number of Iba1-positive cells was significantly reduced in tMCAO-SM (4.22 ± 1.03, * *p* < 0.05) when compared to tMCAO-V (13.50 ± 3.12). In the penumbra area of the striatum, the number of Iba1-positive cells was significantly reduced more in the tMCAO-SM group (10.70 ± 1.48, * *p* < 0.05) than those in the tMCAO-V group (17.03 ± 1.61) (Figure 2C,D).

WB analysis was performed to quantify astrogliosis (Figure 2E–H). In WB, the GFAP, an astrocyte-specific protein, the level was significantly reduced more in the penumbra area of the tMCAO-SM mouse brain (2.41 ± 0.39, * *p* < 0.05) than those in the tMCAO-V group (3.53 ± 0.15) (Figure 2E,F). Moreover, to quantify the level of microgliosis, microglia-specific protein Iba-1 level was significantly reduced in tMCAO-SM (0.83 ± 0.024, **** *p* < 0.001) when compared to tMCAO-V (1.64 ± 0.12). Gliosis was significantly reduced in the tMCAO-SM group compared to that in the tMCAO-V group (Figure 2G,H).

### 3.3. SM Administration Attenuated Inflammation in tMCAO Mouse Brain

**Figure 3:**
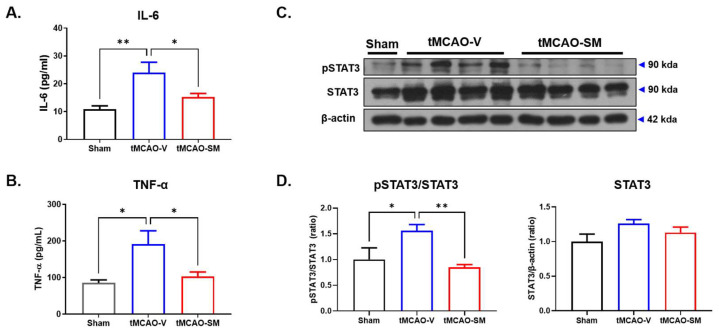
SM reduced inflammation in penumbra area of tMCAO mouse brain. (**A**) Quantification of IL-6 and (**B**) TNF-α level (Sham, *n* = 6; tMCAO-V, *n* = 6; tMCAO-SM, *n* = 6). (**C**) Western blot to confirm STAT3 and pSTAT3. β-actin is a loading control in penumbra area including cortex and striatum. (**D**) Quantification of pSTAT3, and STAT3 level. (Sham, *n* = 4; tMCAO-V, *n* = 6; tMCAO-SM, *n* = 6). Values are expressed as mean ± SEM. Statistical analysis between three groups was performed using the one-way ANOVA following by Tukey’s multiple comparisons test. * *p* < 0.05 and ** p < 0.01.

Glial cells activated by tMCAO secrete inflammatory cytokines that stimulate immune-activating inflammatory transcription factors, such as STAT3 [34]. In previous studies, proinflammatory cytokines such as IL-6 and TNF-α that glial cells secrete, induce inflammation in ischemic stroke [35]. In particular, IL-6 promotes phosphorylation of STAT3 [36]. Therefore, the expression levels of IL-6 and TNF-α in the brain were investigated using ELISA. We found that IL-6 and TNF-α levels were significantly lower in the penumbra area of the tMCAO-SM group (IL-6, 15.3 ± 1.23, * *p* < 0.05; TNF-α, 102.6 ± 12.81, * *p* < 0.05) than those in the tMCAO-V group (IL-6, 24.06 ± 3.67; TNF-α, 191.5 ± 36.3) (Figure 3A,B). Next, the protein levels of STAT3 and pSTAT3 were investigated using WB (Figure 3C). The ratio of pSTAT3/STAT3 was significantly lower in the penumbra area of the tMCAO-SM group (0.85 ± 0.06, ** *p* < 0.01) than that in the tMCAO-V group (1.56 ± 0.12) (Figure 3D).

### 3.4. SM Administration Attenuated Oxidative Stress and Reduced Ferroptosis in tMCAO Mouse Brain

**Figure 4:**
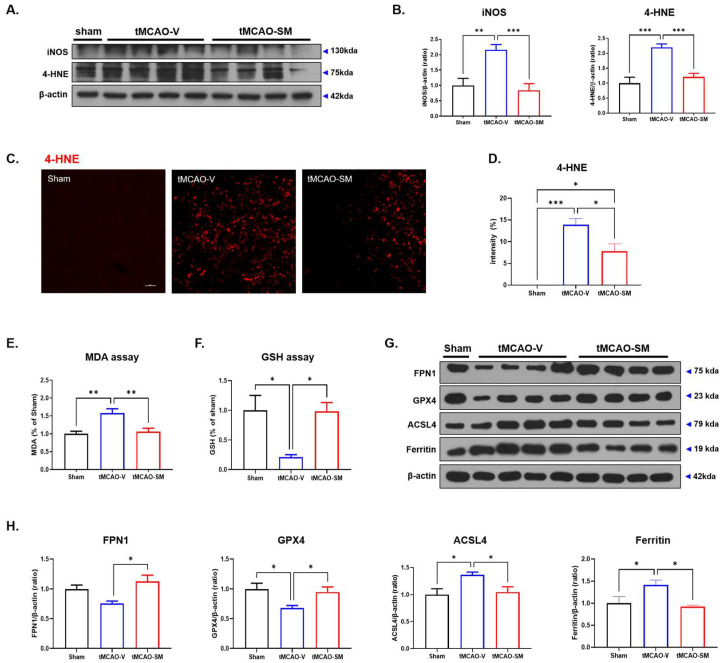
SM alleviated oxidative stress and ferroptosis in tMCAO mouse brain. (**A**) Representative Western blots showing the expression of iNOS and 4-HNE proteins in penumbra region of tMCAO mouse brain. (**B**) Protein level quantification of iNOS and 4-HNE in penumbra region of tMCAO mouse brain. (Sham, *n* = 4; tMCAO-V, *n* = 6; tMCAO-SM, *n* = 6). (**C**) Representative images of 4-HNE staining in penumbra region of tMCAO mouse brain. (**D**) 4-HNE quantification of intensity in tMCAO mice (Sham, *n* = 3; tMCAO-V, *n* = 6; tMCAO-SM, *n* = 6). (**E**) Quantification of MDA level using MDA assay kit (**F**) Quantification of GSH level using GSH assay kit. (**G**) Representative Western blots showing the expression of FPN1, GPX4, ACSL4 and Ferritin proteins. (**H**) Quantification of Western blot for FPN1, GPX4, ACSL4, and Ferritin. (Sham, *n* = 4; tMCAO-V, *n* = 6; tMCAO-SM, *n* = 6). Values are expressed by the mean ± SEM. Statistical analysis between the three groups was performed by the one-way ANOVA following Tukey’s multiple comparisons test. * *p* < 0.05, ** p < 0.01, and *** p < 0.001.

tMCAO-induced I/R injury causes oxidative stress via cellular organelle dysfunction. Reactive oxidative species (ROS) and lipid peroxides can irreversibly damage brain tissue. Therefore, we examined the effect of SM on tMCAO-induced oxidative stress using WB and IHC to determine the iNOS and 4-HNE levels, which are biomarkers of oxidative stress [37]. In the tMCAO-V group (iNOS, 2.16 ± 0.17, ** *p* < 0.01; 4-HNE, 2.19 ± 0.12, *** *p* < 0.001), the levels of iNOS and 4-HNE were significantly higher than those in the sham group in WB (iNOS, 1.00 ± 0.23, 4-HNE, 1.00 ± 0.20) (Figure 4A,B). Interestingly, iNOS and 4-HNE levels were significantly reduced in the tMCAO-SM group in WB (iNOS, 0.83 ± 0.22; 4-HNE, 1.21 ± 0.12, *** *p* < 0.001) (Figure 4B). Additionally, in IHC, the intensity of 4-HNE was significantly lower in the tMCAO-SM group than that in the tMCAO-V group (0.99 ± 0.08, * *p* < 0.05) (Figure 4C,D). Thus, SM reduces oxidative stress in tMCAO mouse brains.

During I/R injury, increased iron levels were observed in the ischemic area. Accumulated oxidative stress and iron promote the induction of injury-mediated cell death, such as ferroptosis [10,38,39]. Therefore, to verify the anti-ferroptotic effects of SM in tMCAO mice, MDA, a biomarker of oxidative stress [40,41], and GSH and GPX4, biomarkers of lipid repair function [42] were investigated using ELISA and WB. In the tMCAO-SM group (1.06 ± 0.09, * *p* <0.05), MDA was significantly reduced compared to the tMCAO-V group (1.58 ± 0.12) (Figure 4E). Conversely, in the tMCAO-SM group (0.98 ± 0.15, * *p* < 0.05), the GSH level was significantly increased compared to the tMCAO-V group (0.21 ± 0.04) (Figure 4F).

Phosphorylated STAT3 disrupts the production of FPN1, which exports iron [11]. Accumulated iron may induce lipid peroxidation and ROS production and worsen ferroptosis-mediated cell death. In the tMCAO-SM group (1.13 ± 0.11, * *p* < 0.05), the FPN1 level was significantly increased compared to the tMCAO-V group (0.75 ± 0.04) (Figure 4G,H). GPX4 neutralizes toxic ROS in the cell membrane to prevent cell death via ferroptosis. The protein level of GPX4 was significantly increased in the tMCAO-SM group (0.95 ± 0.08, * *p* < 0.05) compared to the tMCAO-V group (0.68 ± 0.04) (Figure 4G,H). Acyl-CoA synthetase long-chain family member 4 (ACSL4) is generally considered to be universally required for ferroptosis [43]. The ACSL4 level was decreased in the tMCAO-SM group (1.05 ± 0.10, * *p* < 0.05) compared to that in the tMCAO-V group (1.37 ± 0.05) (Figure 4G,H). Ferritin is a major iron storage protein that plays a vital role in iron metabolism [44]. The ferritin level was decreased more in the tMCAO-SM group (0.92 ± 0.03, * *p* < 0.05) than that in the tMCAO-V group (1.41 ± 0.11) (Figure 4G,H). Next, we conducted Diaminobenzidine(DAB)-Enhanced Perls’ Staining to assess the changes in ferrous iron accumulation in the brains of tMCAO mice. Our findings revealed that the number of iron depositions in the peri-infarct striatum of the tMCAO-V group was significantly higher than the sham-operated groups (tMCAO-V, 3.52 ± 1.70; Sham, 1.0 ± 0.40, *p* < 0.05) (Figure S5). However, we observed that SM administration significantly reduced the iron deposition resulting from cerebral injury in the brains of tMCAO mice (0.94 ± 0.18, *p* < 0.05 vs. tMCAO-V) (Figure S5). Thus, SM had an anti-ferroptotic effect in the tMCAO mouse brain.

### 3.5. SM Administration Induced Synaptic Stability and Reduced Neuronal Loss in tMCAO Mouse Brain

**Figure 5:**
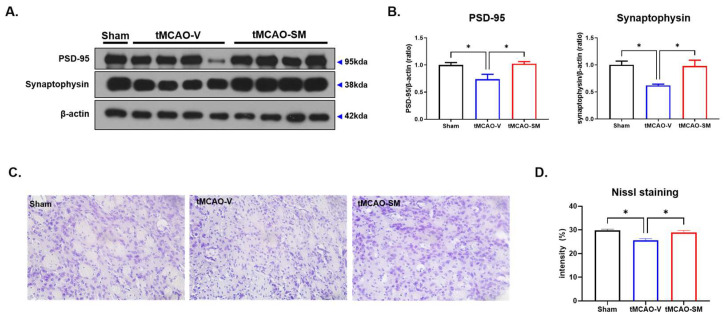
SM reduced synaptic loss and neuronal loss in tMCAO mouse brain. (**A**) Representative Western blots showing the expression of PSD-95 and Synaptophysin proteins in mice brains. (**B**) Quantification level of PSD-95 and synaptophysin proteins. β-actin was a loading control. (PSD-95, Sham, *n* = 4; tMCAO-V, *n* = 4; tMCAO-SM, *n* = 4; Synaptophysin, Sham, *n* = 4; tMCAO-V, *n* = 5; tMCAO-SM, *n* = 5). (**C**) Nissl staining in the penumbra area of the tMCAO mouse brain. Scale bar, 100 μm (**D**) Quantification for neuronal density in the striatum. (Sham, *n* = 3; tMCAO-V, *n* = 5; tMCAO-SM, *n* = 4). Statistical analysis between the three groups was performed by the one-way ANOVA following Tukey’s multiple comparisons test. * *p* < 0.05.

Ischemic stroke induces I/R injury in cerebral tissues. The ROS induced by I/R injury can injure neurons [45]. Therefore, we elucidated the effects of SM on synaptic and neuronal loss in the post-ischemic brain using WB and Nissl staining. Post-synaptic protein PSD-95 was significantly increased in the tMCAO-SM group (1.02 ± 0.04, * *p* < 0.05) compared to that in the tMCAO-V group (0.73 ± 0.09). Similarly, pre-synaptic protein synaptophysin was significantly increased in the tMCAO-SM group (0.97 ± 0.10, * *p < 0.05)* compared to that in the tMCAO-V group (0.62 ± 0.02) (Figure 5A,B).

Furthermore, when the penumbra area of the striatum was examined using Nissl staining, the tMCAO-V group showed a significant decrease in neuronal cells (25.55 ± 0.74) compared to the Sham group (29.79 ± 0.49) (Figure 5C,D). However, the administration of SM led to a reversal of neuronal loss in the tMCAO mouse brain (tMCAO-SM group: 28.96 ± 0.84, * *p* < 0.05) compared to the tMCAO-V group (Figure 5C,D), indicating that SM may be effective in preventing synaptic and neuronal loss in the brains of mice with tMCAO.

### 3.6. Repeated SM Administration Showed Long-Term Neuroprotective Effects against Brain Injures Following tMCAO

**Figure 6:**
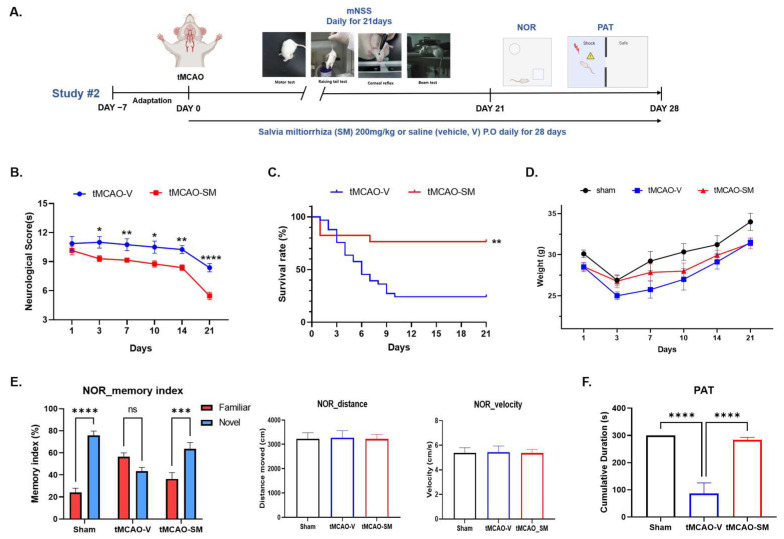
Experiment scheme and SM alleviate memory loss in tMCAO mouse model. (**A**) Experimental scheme. (**B**) Comparing neurological score (mNSS) between tMCAO-V and tMCAO-SM group at 1, 3, 7, 10, 14, 21 days after tMCAO induction (tMCAO-V, *n* = 8; tMCAO-SM, *n* = 13). Values are expressed as the mean ± SEM. Statistical analysis each day between two groups was performed using unpaired *t* test. (**C**) Probability of survival between tMCAO-V and tMCAO-SM group (tMCAO-V, *n* = 33; tMCAO-SM, *n* = 17). Values are expressed as the mean ± SEM. Statistical analysis was performed using Log-rank (Mantel-Cox) test. (**D**) Quantification of recovering body weight. (**E**) Data from NOR test and (**F**) PAT test were analyzed. (Sham, *n* = 9; tMCAO-V, *n* = 8; tMCAO-SM, *n* = 13). Values are expressed as the mean ± SEM. Statistical analysis between the three groups was performed using the two-way ANOVA following Bonferroni’s multiple comparisons test for NOR test and one-way ANOVA following Tukey’s multiple comparisons test for PAT test. * *p* < 0.05, ** p < 0.01, and *** p < 0.001, and **** *p* < 0.0001.

To investigate whether the neuroprotective effects of SM on ischemic brain injuries after tMCAO could be observed in the long term, immediately after tMCAO, we randomly selected animals and administered vehicle or SM daily for 28 days (Figure 6A).

Neurological deficits were assessed daily for 28 days using the neurological score(s) (mNSS) (Figure 6A). Repeated SM administration alleviated neurological deficits more in the tMCAO-SM group (10.15 ± 0.45; 9.30 ± 0.30; 9.15 ± 0.22; 8.76 ± 0.32; 8.38 ± 0.28; and 5.46 ± 0.38; * *p* < 0.05, ** *p* < 0.01, **** *p* < 0.0001) than those in the tMCAO-V group (10.88 ± 0.71; 11.00 ± 0.59; 10.75 ± 0.61; 10.50 ± 0.62; 10.25 ± 0.41; and 8.37 ± 0.41) at 1, 3, 7, 10, 14, 21 days after the tMCAO induction (Figure 6B). We also calculated the probability of survival between the tMCAO-SM and tMCAO-V groups. In the tMCAO-SM group, the survival rate was significantly higher than that in the tMCAO-V group at 28 days (Figure 6C). In terms of body weight, by recording their body weight for 3 weeks, we continuously monitored the body weights of mice in all groups, confirming that the body weights of mice that received sham or tMCAO temporarily decreased after surgery and then gradually returned to normal (Figure 6D).

To investigate the effect of SM on post-stroke cognitive impairment, the NOR and PAT tests were performed 3 weeks after tMCAO (Figure 6A). In the NOR test, sham, tMCAO-V, and tMCAO-SM groups were compared, and significant differences were found in the memory index between sham and tMCAO-V groups and between tMCAO-V and tMCAO-SM groups (sham vs tMCAO-V; 75.88 ± 3.85 vs 43.44 ± 3.42, *** *p* < 0.001; tMCAO-V vs tMCAO-SM, 43.44 ± 3.42 vs 63.61 ± 5.68, * *p* < 0.05) (Figure 6E). However, in the NOR test, the total distance traveled and velocity did not differ among the three groups (Figure 6E). In the PAT, the tMCAO-SM group (283 ± 9.80) shows a similar cumulative duration to the sham group (300 ± 0.00), but the tMCAO-V group showed a relatively low cumulative duration (Figure 6F). There was a significant difference between the tMCAO-SM (283 ± 9.80, **** *p* < 0.0001) and tMCAO-V groups (86.38 ± 39.21) (Figure 6F). Repeated SM administration alleviated cognitive impairment in the tMCAO mouse model.

## 4. Discussion

Ischemic stroke induces oxidative stress, inflammation, and I/R injury, which damage cellular organelles in the brain [46]. Recently, interest in crude herbal extracts that protect against cerebral I/R injury has increased [47]. Herbal extracts with relatively high stability and few side effects even when used for a long time are ideal. Various extracts that protect against cerebral I/R injury have been studied in tMCAO models. For example, in the tMCAO model, Coicis Semen is a herb that alleviates oxidative stress via the TGF-β, ALK1, and Smad1,5 signaling pathway [48]. Additionally, flaxseed oil promotes the recovery of motor function via the promotion of neurotrophic factors [49].

SM, also known as Danshen, is a traditional Chinese herb that has been used for centuries in the treatment of various ailments, including cardiovascular diseases and cerebrovascular diseases [24,50]. It contains acitve compounds such as salvianolic acid B, tanshinone IIA, and cryptotanshinone, which are believed to have antioxidant, anti-inflammatory, and anti-apoptotic effects, and have been shown to have neuroprotective effects in animal models of stroke [51,52,53]. These compounds have been found to improve cerebral blood flow, reduce oxidative stress, and inhibit inflammation, thereby protecting neurons from damage and promoting their survival [23]. The therapeutic mechanisms underlying the neuroprotective effects of SM are still being investigated, but its potential as a treatment for stroke and other neurological disorders is promising [24].

This study showed that SM showed promising short- (3 days) and long-term (28 days) neuroprotective effects in tMCAO mice. SM administration reduced the infarct volume in the acute phase of tMCAO using MRI with T2-weighted imaging 3 days after tMCAO in all groups; these results were confirmed by TTC staining. The patterns of metabolite distribution in the brains of both tMCAO groups were analyzed using MRS within 3 days after onset. Concentrations of Tau, tCr, and Glx were significantly higher in the tMCAO-SM group than those in the tMCAO-V group. Tau and tCr may have helped alleviate I/R injury, whereas Glx may have helped in memory loss recovery. In addition, SM administration attenuated acute ischemic brain injuries, such as brain infarction and functional neurological deficits, in tMCAO mice. After a stroke, reactive gliosis and glial scars form in the lesion area [54]. Although these changes are conventionally considered to play an important role in the clearance of neuronal debris during the early stages of stroke, a recent report demonstrated that reactive microglia and astrocytes hinder brain repair by engulfing synapses and inhibiting phagocytosis before glial scar maturation [55]. Additionally, microglia in the penumbra area of the tMCAO brain are rapidly activated after I/R injury and release inflammatory cytokines, including TNF-α, that induce neuronal tissue injury [34,56]. Consistent with our results, we confirmed that SM treatment normalized the reactive gliosis as well as pro-inflammatory cytokines, including the IL-6 and TNF-α, in the penumbra area of tMCAO brain. In addition, following treatment with SM, the protein levels of pSTAT3/STAT3 and immune-activating inflammatory transcription factors were ameliorated in tMCAO mice. Because I/R injury promotes the secretion of IL-6, which induces phosphorylation of STAT3 and blocks the level of FPN1 protein [11], accumulated iron causes lipid peroxidation, which damages the cellular membrane [57], upregulates toxic ROS accumulation, such as iNOS [58,59] and increases lethal lipid peroxides, such as 4-HNE and MDA, in an ischemic stroke mouse model [40,41,60,61,62].

Ferroptosis, which induces disturbances in brain iron homeostasis, is considered a mechanism of acute neuronal cell death following ischemic stroke [63]. Furthermore, ferroptotic responses related to oxidative stress, amino acid metabolism, and lipid metabolism can be regulated by inducing or inhibiting redox- or iron metabolism-related proteins [64]. Accumulation of lipid peroxide and ROS triggers ferroptosis [65].

In our results, the levels of oxidative stress, including lipid peroxidation products, such as MDA, 4-HNE, and ACSL4, were increased in the penumbra of tMCAO mice and were recovered by SM treatment. In particular, ACSL4 is an important isozyme for polyunsaturated fatty acid metabolism and exacerbates ischemic brain injury by enhancing lipid peroxidation, ferroptosis, and microglia-mediated neuroinflammation [66]. SM treatment in tMCAO mice upregulated GPX4, a key anti-lipid peroxidation enzyme, and GSH, a cofactor that reduces peroxides via GPX4 [67,68]. In previous studies, protein levels of ferroportin 1 (FPN1), an Fe^2+^ exporter in the extracellular space, were downregulated, and ferritin, which maintains the equilibrium of iron, was upregulated in the ischemic brain of mice. SM administration also dramatically attenuated the dysregulation of iron metabolism-related proteins, such as FPN1 and ferritin. These results reflected the level of ferroptosis in the brains of tMCAO mice. Our results show that SM treatment alleviates ferroptotic cell death by upregulating lipid peroxidation during cerebral I/R injury.

Stroke is a major cause of long-term physical disabilities in adults. Cognitive impairment and memory dysfunction are common after stroke diagnosis and lead to post-stroke dementia [69]. Clinically, approximately 30% of stroke patients develop dementia within 1 year of stroke onset [70]. Thus, we investigated whether SM could also have long-term neuroprotective effects against brain injury after tMCAO. Notably, this study demonstrated that regular SM administration for 28 days could provide long-term neuroprotective effects in tMCAO. In the NOR and PAT tests, tMCAO mice showed cognitive impairment; however, long-term SM administration ameliorated memory loss in tMCAO mice. In the tMCAO-SM group, synaptic proteins, such as PSD-95 and synaptophysin, recovered as much as in the sham group, indicating that synaptic loss, memory impairment, and neurological deficits were alleviated.

Our study demonstrates that the administration of SM can effectively reduce brain damage caused by ferrotic cell death induced by dysregulation of oxidative stress as well as upregulation of inflammatory response. Notably, repeated administration of SM significantly improved cognitive impairment, reduced neurological deficits, and increased survival rate. These results suggest that SM may have the potential to be a viable alternative for the treatment of ischemic stroke as a neuroprotective agent.

## Data Availability

The data supporting the findings of this study are available from the corresponding author upon reasonable request.

## Supplementary Materials

The following are available online at https://www.mdpi.com/article/10.3390/antiox12040785/s1, Figure S1: Blood flow tracking by Laser doppler blood flowmeter, Figure S2: Analysis of SM using High-Performance Liquid Chromatography (HPLC), Figure S3: Quantification of brain edema in tMCAO-V and tMCAO-SM groups on day 3, Figure S4: SM alleviates cerebral I/R injury in MRI T2 imaging on day 10, Figure S5: SM reduced iron accumulation in tMCAO mouse brain. Reference [71] is cited in Supplementary Materials.

## References

[1] Campbell B.C.V., De Silva D.A., Macleod M.R., Coutts S.B., Schwamm L.H., Davis S.M., Donnan G.A. (2019). Ischaemic stroke. Nat. Rev. Dis. Prim..

[2] Feigin V.L., Brainin M., Norrving B., Martins S., Sacco R.L., Hacke W., Fisher M., Pandian J., Lindsay P. (2022). World Stroke Organization (WSO): Global Stroke Fact Sheet 2022. Int. J. Stroke.

[3] Feigin V.L., Stark B.A., Johnson C.O., Roth G.A., Bisignano C., Abady G.G., Abbasifard M., Abbasi-Kangevari M., Abd-Allah F., Abedi V. (2021). Global, regional, and national burden of stroke and its risk factors, 1990–2019: A systematic analysis for the Global Burden of Disease Study 2019. Lancet Neurol..

[4] Herpich F., Rincon F. (2020). Management of Acute Ischemic Stroke. Crit. Care Med..

[5] Lo J.W., Crawford J.D., Desmond D.W., Godefroy O., Jokinen H., Mahinrad S., Bae H.J., Lim J.S., Kohler S., Douven E. (2019). Profile of and risk factors for poststroke cognitive impairment in diverse ethnoregional groups. Neurology.

[6] Aqueveque P., Ortega P., Pino E., Saavedra F., Germany E., Gómez B. (2017). After Stroke Movement Impairments: A Review of Current Technologies for Rehabilitation. Physical Disabilities—Therapeutic Implications.

[7] Moskowitz M.A., Lo E.H., Iadecola C. (2010). The science of stroke: Mechanisms in search of treatments. Neuron.

[8] Liu Y., Fang Y., Zhang Z., Luo Y., Zhang A., Lenahan C., Chen S. (2022). Ferroptosis: An emerging therapeutic target in stroke. J. Neurochem..

[9] Zhang Y., Lu X., Tai B., Li W., Li T. (2021). Ferroptosis and Its Multifaceted Roles in Cerebral Stroke. Front. Cell Neurosci..

[10] Ren J.X., Li C., Yan X.L., Qu Y., Yang Y., Guo Z.N. (2021). Crosstalk between Oxidative Stress and Ferroptosis/Oxytosis in Ischemic Stroke: Possible Targets and Molecular Mechanisms. Oxid. Med. Cell Longev..

[11] Fang X.L., Ding S.Y., Du X.Z., Wang J.H., Li X.L. (2022). Ferroptosis-A Novel Mechanism With Multifaceted Actions on Stroke. Front. Neurol..

[12] Pan Y., Wang X., Liu X., Shen L., Chen Q., Shu Q. (2022). Targeting Ferroptosis as a Promising Therapeutic Strategy for Ischemia-Reperfusion Injury. Antioxidants.

[13] Wei Z., Xie Y., Wei M., Zhao H., Ren K., Feng Q., Xu Y. (2022). New insights in ferroptosis: Potential therapeutic targets for the treatment of ischemic stroke. Front. Pharmacol..

[14] Lalkovicova M., Danielisova V. (2016). Neuroprotection and antioxidants. Neural. Regen. Res..

[15] Yang K., Zeng L., Yuan X., Wang S., Ge A., Xu H., Zeng J., Ge J. (2022). The mechanism of ferroptosis regulating oxidative stress in ischemic stroke and the regulation mechanism of natural pharmacological active components. Biomed. Pharmacother..

[16] Liu X., Du Y., Liu J., Cheng L., He W., Zhang W. (2023). Ferrostatin-1 alleviates cerebral ischemia/reperfusion injury through activation of the AKT/GSK3beta signaling pathway. Brain Res. Bull..

[17] Bahonar A., Saadatnia M., Khorvash F., Maracy M., Khosravi A. (2017). Carotenoids as Potential Antioxidant Agents in Stroke Prevention: A Systematic Review. Int. J. Prev. Med..

[18] Shirley R., Ord E.N., Work L.M. (2014). Oxidative Stress and the Use of Antioxidants in Stroke. Antioxidants.

[19] Zhou R., He L.F., Li Y.J., Shen Y., Chao R.B., Du J.R. (2012). Cardioprotective effect of water and ethanol extract of Salvia miltiorrhiza in an experimental model of myocardial infarction. J. Ethnopharmacol..

[20] Su C.Y., Ming Q.L., Rahman K., Han T., Qin L.P. (2015). Salvia miltiorrhiza: Traditional medicinal uses, chemistry, and pharmacology. Chin. J. Nat. Med..

[21] Lv H., Wang L., Shen J., Hao S., Ming A., Wang X., Su F., Zhang Z. (2015). Salvianolic acid B attenuates apoptosis and inflammation via SIRT1 activation in experimental stroke rats. Brain Res. Bull..

[22] Yu X.Y., Lin S.G., Zhou Z.W., Chen X., Liang J., Duan W., Yu X.Q., Wen J.Y., Chowbay B., Li C.G. (2007). Tanshinone IIB, a primary active constituent from Salvia miltiorrhza, exhibits neuro-protective activity in experimentally stroked rats. Neurosci. Lett..

[23] Lin T.H., Hsieh C.L. (2010). Pharmacological effects of Salvia miltiorrhiza (Danshen) on cerebral infarction. Chin. Med..

[24] Meim X.-D., Cao Y.F., Che Y.Y., Li J., Shang Z.P., Zhao W.J., Qiao Y.J., Zhang J.Y. (2019). Danshen: A phytochemical and pharmacological overview. Chin. J. Nat. Med..

[25] Dirnagl U., Iadecola C., Moskowitz M.A. (1999). Pathobiology of ischaemic stroke: An integrated view. Trends Neurosci..

[26] Wang C., Zhao R., Li B., Gu L.Y., Gou H. (2016). An in vivo and in vitro study: High-dosage Danshen injection induces peripheral vascular endothelial cells injury. Hum. Exp. Toxicol..

[27] Chen J., Sanberg P.R., Li Y., Wang L., Lu M., Willing A.E., Sanchez-Ramos J., Chopp M. (2001). Intravenous administration of human umbilical cord blood reduces behavioral deficits after stroke in rats. Stroke.

[28] Hennig J., Nauerth A., Friedburg H. (1986). RARE imaging: A fast imaging method for clinical MR. Magn. Reson. Med..

[29] Bottomley P.A. (1987). Spatial localization in NMR spectroscopy in vivo. Ann. N. Y. Acad. Sci..

[30] Tkac I., Starcuk Z., Choi I.Y., Gruetter R. (1999). In vivo 1H NMR spectroscopy of rat brain at 1 ms echo time. Magn. Reson. Med..

[31] Provencher S.W. (1982). A constrained regularization method for inverting data represented by linear algebraic or integral equations. Comput. Phys. Commun..

[32] Kim J.H., Lim D.K., Suh Y.H., Chang K.A. (2021). Long-Term Treatment of Cuban Policosanol Attenuates Abnormal Oxidative Stress and Inflammatory Response via Amyloid Plaques Reduction in 5xFAD Mice. Antioxidants.

[33] del Zoppo G., Ginis I., Hallenbeck J.M., Iadecola C., Wang X., Feuerstein G.Z. (2000). Inflammation and stroke: Putative role for cytokines, adhesion molecules and iNOS in brain response to ischemia. Brain Pathol..

[34] Xu S., Lu J., Shao A., Zhang J.H., Zhang J. (2020). Glial Cells: Role of the Immune Response in Ischemic Stroke. Front. Immunol..

[35] Tuttolomondo A., Di Raimondo D., di Sciacca R., Pinto A., Licata G. (2008). Inflammatory cytokines in acute ischemic stroke. Curr. Pharm. Des..

[36] Zhu H., Hu S., Li Y., Sun Y., Xiong X., Hu X., Chen J., Qiu S. (2022). Interleukins and Ischemic Stroke. Front. Immunol..

[37] Li Z., Bi R., Sun S., Chen S., Chen J., Hu B., Jin H. (2022). The Role of Oxidative Stress in Acute Ischemic Stroke-Related Thrombosis. Oxid. Med. Cell Longev..

[38] Reichert C.O., de Freitas F.A., Sampaio-Silva J., Rokita-Rosa L., Barros P.L., Levy D., Bydlowski S.P. (2020). Ferroptosis Mechanisms Involved in Neurodegenerative Diseases. Int. J. Mol. Sci..

[39] Xie Y., Hou W., Song X., Yu Y., Huang J., Sun X., Kang R., Tang D. (2016). Ferroptosis: Process and function. Cell Death Differ..

[40] Lee W.C., Wong H.Y., Chai Y.Y., Shi C.W., Amino N., Kikuchi S., Huang S.H. (2012). Lipid peroxidation dysregulation in ischemic stroke: Plasma 4-HNE as a potential biomarker?. Biochem. Biophys. Res. Commun..

[41] Bir L.S., Demir S., Rota S., Koseoglu M. (2006). Increased serum malondialdehyde levels in chronic stage of ischemic stroke. Tohoku J. Exp. Med..

[42] Ursini F., Maiorino M. (2020). Lipid peroxidation and ferroptosis: The role of GSH and GPx4. Free Radic. Biol. Med..

[43] Yuan H., Li X., Zhang X., Kang R., Tang D. (2016). Identification of ACSL4 as a biomarker and contributor of ferroptosis. Biochem. Biophys. Res. Commun..

[44] Arosio P., Levi S. (2002). Ferritin, iron homeostasis, and oxidative damage. Free Radic. Biol. Med..

[45] Bu Z.Q., Yu H.Y., Wang J., He X., Cui Y.R., Feng J.C., Feng J. (2021). Emerging Role of Ferroptosis in the Pathogenesis of Ischemic Stroke: A New Therapeutic Target?. ASN Neuro.

[46] Chamorro A., Dirnagl U., Urra X., Planas A.M. (2016). Neuroprotection in acute stroke: Targeting excitotoxicity, oxidative and nitrosative stress, and inflammation. Lancet Neurol..

[47] Tao T., Liu M., Chen M., Luo Y., Wang C., Xu T., Jiang Y., Guo Y., Zhang J.H. (2020). Natural medicine in neuroprotection for ischemic stroke: Challenges and prospective. Pharmacol. Ther..

[48] Du J., Yin G., Hu Y., Shi S., Jiang J., Song X., Zhang Z., Wei Z., Tang C., Lyu H. (2020). Coicis semen protects against focal cerebral ischemia-reperfusion injury by inhibiting oxidative stress and promoting angiogenesis via the TGFbeta/ALK1/Smad1/5 signaling pathway. Aging.

[49] Bagheri A., Talei S., Hassanzadeh N., Mokhtari T., Akbari M., Malek F., Jameie S.B., Sadeghi Y., Hassanzadeh G. (2017). The Neuroprotective Effects of Flaxseed Oil Supplementation on Functional Motor Recovery in a Model of Ischemic Brain Stroke: Upregulation of BDNF and GDNF. Acta Med. Iran..

[50] Mahalakshmi B., Huang C.-Y., Lee S.-D., Maurya N., Kiefer R., Bharath Kumar V. (2021). Review of Danshen: From its metabolism to possible mechanisms of its biological activities. J. Funct. Foods.

[51] Li X., Guo K., Zhang R., Wang W., Sun H., Yague E., Hu Y. (2022). Exploration of the Mechanism of Salvianolic Acid for Injection Against Ischemic Stroke: A Research Based on Computational Prediction and Experimental Validation. Front. Pharmacol..

[52] Song Z., Feng J., Zhang Q., Deng S., Yu D., Zhang Y., Li T. (2021). Tanshinone IIA Protects Against Cerebral Ischemia Reperfusion Injury by Regulating Microglial Activation and Polarization via NF-kappaB Pathway. Front. Pharmacol..

[53] Zhu F., Chen H., Xu M., Zhang X., Yu J., Pan Y., Zhu W. (2021). Cryptotanshinone possesses therapeutic effects on ischaemic stroke through regulating STAT5 in a rat model. Pharm. Biol..

[54] Wanner I.B., Anderson M.A., Song B., Levine J., Fernandez A., Gray-Thompson Z., Ao Y., Sofroniew M.V. (2013). Glial scar borders are formed by newly proliferated, elongated astrocytes that interact to corral inflammatory and fibrotic cells via STAT3-dependent mechanisms after spinal cord injury. J. Neurosci..

[55] Shi X., Luo L., Wang J., Shen H., Li Y., Mamtilahun M., Liu C., Shi R., Lee J.H., Tian H. (2021). Stroke subtype-dependent synapse elimination by reactive gliosis in mice. Nat. Commun..

[56] Gulke E., Gelderblom M., Magnus T. (2018). Danger signals in stroke and their role on microglia activation after ischemia. Ther. Adv. Neurol. Disord..

[57] Jiang X., Stockwell B.R., Conrad M. (2021). Ferroptosis: Mechanisms, biology and role in disease. Nat. Rev. Mol. Cell Biol..

[58] Yuan Z., Liu W., Liu B., Schnell A., Liu K.J. (2010). Normobaric hyperoxia delays and attenuates early nitric oxide production in focal cerebral ischemic rats. Brain Res..

[59] Zhao Y., Huang Y., Fang Y., Zhao H., Shi W., Li J., Duan Y., Sun Y., Gao L., Luo Y. (2018). Chrysophanol attenuates nitrosative/oxidative stress injury in a mouse model of focal cerebral ischemia/reperfusion. J. Pharmacol. Sci..

[60] Liu X., Bai M., Fan L., Lou Z. (2022). Serum 4-hydroxynonenal associates with the recurrence of patients with primary cerebral infarction. Front. Cell Neurosci..

[61] Chen H., Yoshioka H., Kim G.S., Jung J.E., Okami N., Sakata H., Maier C.M., Narasimhan P., Goeders C.E., Chan P.H. (2011). Oxidative stress in ischemic brain damage: Mechanisms of cell death and potential molecular targets for neuroprotection. Antioxid. Redox Signal..

[62] Lorente L., Martin M.M., Abreu-Gonzalez P., Ramos L., Argueso M., Sole-Violan J., Riano-Ruiz M., Jimenez A. (2015). Serum malondialdehyde levels in patients with malignant middle cerebral artery infarction are associated with mortality. PLoS ONE.

[63] Kondo Y., Ogawa N., Asanuma M., Ota Z., Mori A. (1995). Regional differences in late-onset iron deposition, ferritin, transferrin, astrocyte proliferation, and microglial activation after transient forebrain ischemia in rat brain. J. Cereb. Blood Flow Metab..

[64] Chen X., Comish P.B., Tang D., Kang R. (2021). Characteristics and Biomarkers of Ferroptosis. Front. Cell Dev. Biol..

[65] Yan N., Zhang J.J. (2019). The Emerging Roles of Ferroptosis in Vascular Cognitive Impairment. Front. Neurosci..

[66] Cui Y., Zhang Y., Zhao X., Shao L., Liu G., Sun C., Xu R., Zhang Z. (2021). ACSL4 exacerbates ischemic stroke by promoting ferroptosis-induced brain injury and neuroinflammation. Brain Behav. Immun..

[67] Galaris D., Barbouti A., Pantopoulos K. (2019). Iron homeostasis and oxidative stress: An intimate relationship. Biochim. Biophys. Acta Mol. Cell Res..

[68] Ding H., Yan C.Z., Shi H., Zhao Y.S., Chang S.Y., Yu P., Wu W.S., Zhao C.Y., Chang Y.Z., Duan X.L. (2011). Hepcidin is involved in iron regulation in the ischemic brain. PLoS ONE.

[69] Sun J.H., Tan L., Yu J.T. (2014). Post-stroke cognitive impairment: Epidemiology, mechanisms and management. Ann. Transl. Med..

[70] Cullen B., O’Neill B., Evans J.J., Coen R.F., Lawlor B.A. (2007). A review of screening tests for cognitive impairment. J. Neurol. Neurosurg. Psychiatry.

[71] Bao W.D., Pang P., Zhou X.T., Hu F., Xiong W., Chen K., Wang J., Wang F., Xie D., Hu Y.Z. (2021). Loss of ferroportin induces memory impairment by promoting ferroptosis in Alzheimer’s disease. Cell Death Differ..

