# Supplementary material for: Salvia miltiorrhiza Alleviates Memory Deficit Induced by Ischemic Brain Injury in a Transient MCAO Mouse Model by Inhibiting Ferroptosis
Source: Antioxidants (Basel). 2023 Mar 23;12(4):785. doi: 10.3390/antiox12040785 (PMC10135292; doi:10.3390/antiox12040785)
Supplement: Supplementary file 1 [file antioxidants-12-00785-s001.zip › antioxidants-2266541-supplementary.pdf]

Salvia miltiorrhiza Alleviates Memory Deficit Induced by Ischemic Brain Injury in a Transient MCAO Mouse Model by Inhibiting Ferroptosis

Geon Ko [1], Jinho Kim [1], Yeong-Jae Jeon [1], Donghun Lee [2], Hyeon-Man Baek [1,3,4,*] and Keun-A Chang [1,4,5,*]

[1] Department of Health Sciences and Technology, GAIHST, Gachon University, Incheon 21999, Republic of Korea; sirius9725@gachon.ac.kr (G.K.); qpdlzhfl@gachon.ac.kr (J.K.); yeong@gachon.ac.kr (Y.-J.J.)
[2] Department of Herbal Pharmacology, College of Korean Medicine, Gachon University, Seongnam-si 13120, Republic of Korea; dlee@gachon.ac.kr
[3] Department of Molecular Medicine, College of Medicine, Gachon University, Incheon 21999, Republic of Korea
[4] Department of Pharmacology, College of Medicine, Gachon University, Incheon 21999, Republic of Korea
[5] Department of Basic Neuroscience, Neuroscience Research Institute, Gachon University, Incheon 21999, Republic of Korea
* Correspondence: hmbaek98@gachon.ac.kr (H.-M.B.); keuna705@gachon.ac.kr (K.-A.C.); Tel.: +82-32-899-6678 (H.-M.B.); +82-32-899-6411 (K.-A.C.)

Supplementary Material & Methods

High-Performance Liquid Chromatography (HPLC)

Chromatographic analysis of SM was performed by the HPLC linked using an 1100 series HPLC system (Agilent, USA). Chromatographic separation was carried out at 20℃ using a Zorbax EclipseXDB C18 column (4.6 x 250 mm, 5 μm, Agilent, USA). A 10 mg sample was diluted with 1 ml of 100% methanol and then, sonicated for 10 min. Samples were filtered out using a 0.2 μm syringe filter (Waters Corp., USA). The mobile phase component contained 0.1% formic acid (A) and acetonitrile (B) and the column was flowed out as follows: 0-10 min, 10-20%; 10-27 min, 20-33%; 27-30 min, 33-70%; 30-50 min, 85% solvent (B). A 10 μL injection volume was used to mark the run-off at 280 nm.

Quantification of brain edema

After TTC staining, brain edema was quantified by analyzing the area of four slices of brain tissues from the contralateral and ipsilateral hemispheres of both the tMCAO-V and tMCAO-SM groups using image J 1.50 software. The brain edema (%) was calculated as [[(ipsilateral hemisphere-contralateral hemisphere)/contralateral hemisphere] (%) – 100].

Diaminobenzidine- (DAB-) Enhanced Perls' Staining

Perls' staining was performed as previously described [1]. Briefly, brain tissues were washed 3 times with PBS (phosphate-buffered saline) for 5mins each. Then, brain sections were incubated in the Perls' staining solution (5% potassium ferrocyanide [sigma Aldrich, ST. Louis, MO, USA] + 10% hydrochloric acid [sigma Aldrich]) for 1 hours. After washing 3 times (5 min each) with PBS, endogenous peroxidase activity was quenched for 20 min at room temperature in 0.3% H2O2 in methanol, followed by washing with PBS five times. Brain sections were stained with DAB (3,3′-diaminobenzidine) Horseradish Peroxidase for 3 min. Brain sections were placed on a glass slide. A cover glass was mounted on the sections.

Supplementary figures

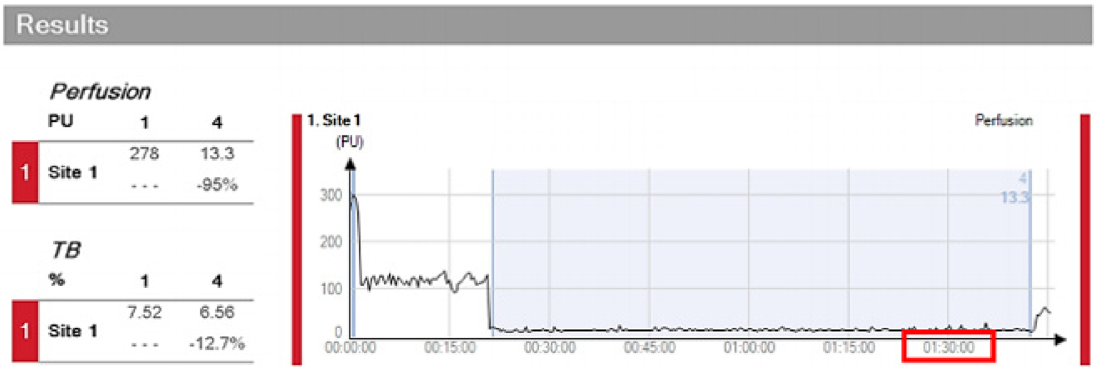

Figure S1. Blood flow tracking by Laser doppler blood flowmeter

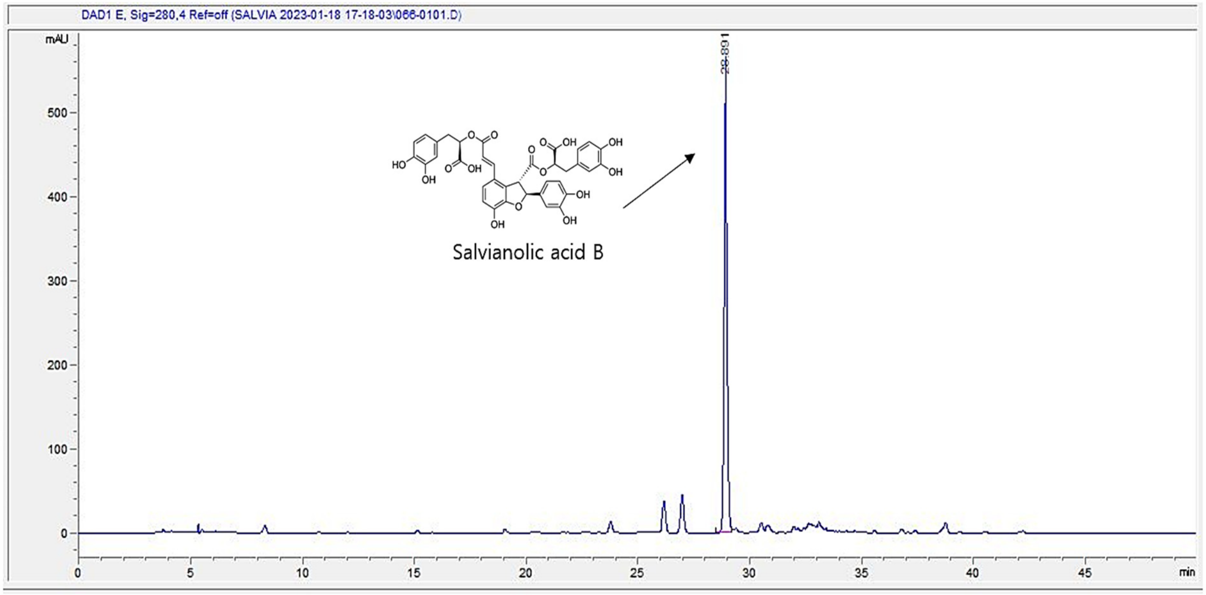

Figure S2. Analysis of SM using High-Performance Liquid Chromatography (HPLC).

10 mg sample was diluted in 1 ml of methanol and sonicated for 10 min. Samples were filtered through a 0.2 µm syringe filter (Waters Corp., USA). The mobile phase composition was 0.1% formic acid (A) and acetonitrile (B) and the column was eluted as follows: 0–10 min, 10-20%; 10–27 min, 20-33%; 27–30 min, 33-70%; 30-50 min, 85% solvent (B) with a flow rate of 1.0 ml/min. The outflow was indicated at 280 nm using an injection volume of 10 µL.

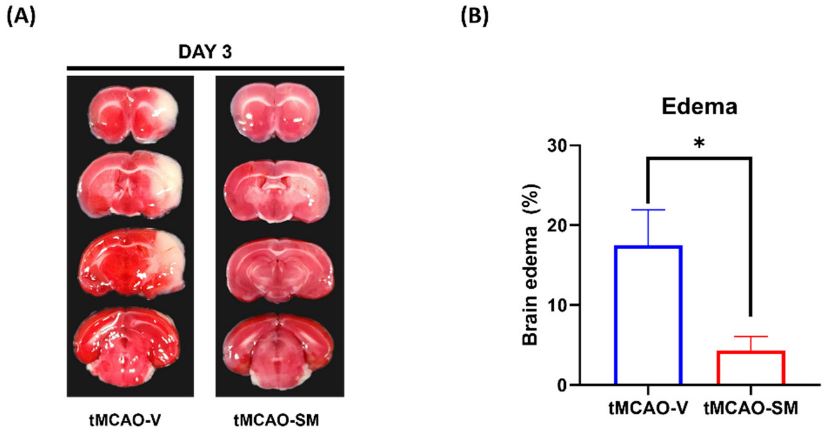

Figure S3. Quantification of brain edema in tMCAO-V and tMCAO-SM groups on day 3

(A) TTC staining and (B) brain edema ratio (%) was compared between tMCAO-V and tMCAO-SM groups, 3 days after tMCAO induction. Statistical analysis between the two groups was performed using unpaired t test. *$p<0.05$.

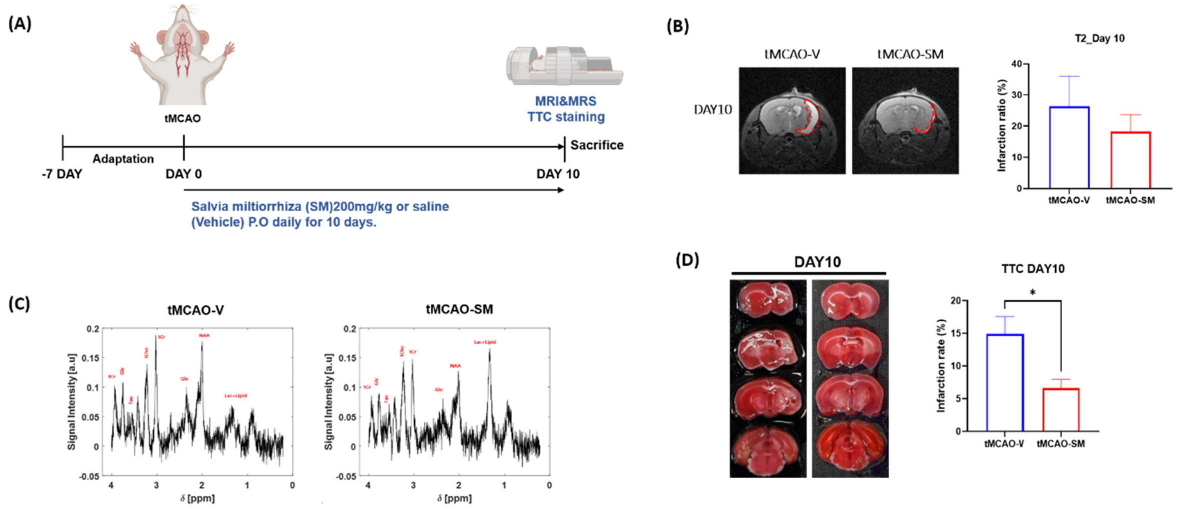

Figure S4. SM alleviates cerebral I/R injury in MRI T2 imaging on day 10.

(A) Experimental scheme. After inducing tMCAO, mice were orally administered the saline or SM for 10 days, tMCAO mice were taken MRI T2 imaging and MRS to analyze how much cerebral I/R injury occurred and recovered. (B) MRI T2 imaging and (C) MRS analysis in tMCAO-V and tMCAO-SM groups on day10. (D) TTC staining and quantification of infarction ratio between tMCAO-V and tMCAO-SM groups 10 days after tMCAO. (tMCAO-V, n=5-6; tMCAO-SM, n=4-7). Statistical analysis between the two groups was performed using unpaired t test. *$p<0.05$.

(A)

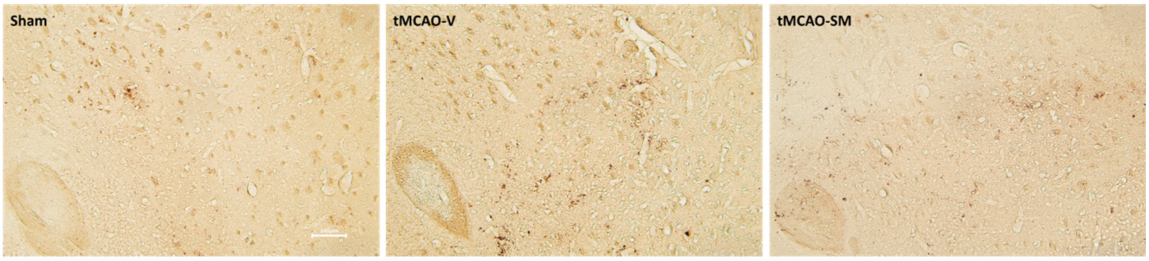

(B)

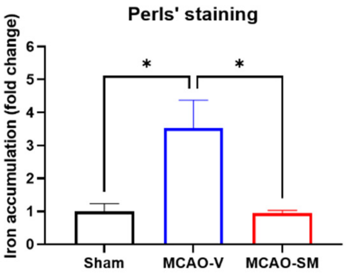

Figure S5. SM reduced iron accumulation in tMCAO mouse brain.

(A) Representative image of Perls' staining in Sham, tMCAO-V, tMCAO-SM groups. Scale bar, 100 μm (B) Quantification of iron accumulation in penumbra area of striatum of the tMCAO mouse brain by DAB-enhanced Perls' staining. (Sham, n=3; tMCAO-V, n=4; tMCAO-SM, n=5) Statistical analysis between the three groups was performed using the one-way ANOVA followed by Tukey's multiple comparisons test. *$p<0.05$.

Reference

1. Bao, W.D.; Pang, P.; Zhou, X.T.; Hu, F.; Xiong, W.; Chen, K.; Wang, J.; Wang, F.; Xie, D.; Hu, Y.Z.; et al. Loss of ferroportin induces memory impairment by promoting ferroptosis in Alzheimer's disease. *Cell Death Differ* **2021**, *28*, 1548-1562, doi:10.1038/s41418-020-00685-9.
